# Inference of population structure using multilocus genotype data: dominant markers and null alleles

**DOI:** 10.1111/j.1471-8286.2007.01758.x

**Published:** 2007-07-01

**Authors:** DANIEL FALUSH, MATTHEW STEPHENS, JONATHAN K PRITCHARD

**Affiliations:** * Peter Medawar Building for Pathogen Research, University of Oxford, Oxford OX1 3SY UK; † Department of Human Genetics, University of Chicago 920 East 58th Street — CLSC 507 Chicago, IL 60637, USA

**Keywords:** admixture, AFLP, polyploids

## Abstract

Dominant markers such as amplified fragment length polymorphisms (AFLPs) provide an economical way of surveying variation at many loci. However, the uncertainty about the underlying genotypes presents a problem for statistical analysis. Similarly, the presence of null alleles and the limitations of genotype calling in polyploids mean that many conventional analysis methods are invalid for many organisms. Here we present a simple approach for accounting for genotypic ambiguity in studies of population structure and apply it to AFLP data from whitefish. The approach is implemented in the program structure version *2.2,* which is available from http://pritch.bsd.uchicago.edu/structure.html.

## Introduction

Methods based on variable polymerase chain reaction (PCR) amplification such as amplified fragment length polymorphisms (AFLPs) can provide a rapid and affordable approach to collecting polymorphism data on a genomic scale (Campbell *et al*. 2003; Luikart *et al*. 2003). However, these markers are typically ambiguous about the genotypes that underlie them. In particular, in diploids, a band will be obtained if either or both of the homologous chromosomes contain an amplifiable sequence. In polyploids, there can be ambiguity even with codominant markers. Even when it is possible to determine which alleles are present, it might be difficult to determine the number of each. These ambiguities need to be addressed in any analysis (e.g. Holsinger *et al*. 2002; Hardy 2003; Hill & Weir 2004; Hollingsworth & Ennos 2004; Kosman & Leonard 2005).

The program structure uses a Markov chain Monte Carlo (MCMC) algorithm to cluster individuals into populations on the basis of multilocus genotype data (Pritchard *et al*. 2000; Falush *et al*. 2003b), and it has been applied to problems such as identifying cryptic population structure, detecting migrants or admixed individuals, and inferring historical population admixture (e.g. Rosenberg *et al*. 2002; Falush *et al*. 2003a; Albert *et al*. 2006; Lecis *et al*. 2006; Ostrowski *et al*. 2006). We describe here an MCMC algorithm within the structure framework that accounts appropriately for the genotypic ambiguity inherent in dominant markers given that other structure assumptions are met (see Model description below). The algorithm is implemented in the newest release of structure (version 2.2, to be made available at http://pritch.bsd.uchicago.edu/), with the result that all available model options can now be applied to data sets that include dominant markers. The approach should also be applicable to a wide range of other MCMC analysis methods.

## Model description

We encourage readers who are unfamiliar with structure to consult Pritchard *et al*. (2000) before proceeding further. In brief, structure assumes that all of the genetic material of the sampled individuals comes from one or more of *K* unobserved populations. Each population is characterized by a set of allele frequencies *P* at each locus, where *P* is a multidimensional vector with elements *p*_*klj*_ representing the frequency of allele *j* at locus *l* in population *k*.

The simplest ancestry model (the no-admixture model) assumes that all of the genetic material from any given individual comes from one population, but more complex models (the admixture, migration, and linkage models) allow for the possibility that individuals may have mixed ancestry in more than one of the *K* populations. When individuals have mixed ancestry, this means that each genotyped allele comes from one or other of the *K* populations. In that case, 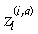 denotes the population of origin of allele copy *a* of individual *i* at locus *l* (we use the term ‘allele copy’ to refer to one of the two alleles carried at a particular locus by a particular individual).

The gene frequencies (represented collectively by *P*) and the population of origin of each allele copy of each individual (represented collectively by *Z*) are assumed to be unknown, so that they must be estimated from the data. However, the previous versions of structure have assumed that the genotype of each individual at each locus is known (or entirely unknown, in the case of missing data). The genotype of allele copy *a* of individual *i* at locus *l* is represented 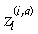. For notational convenience, genotypes are represented as being ordered, even if no phasing information is available, so that for a diploid, 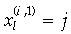,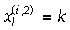 is distinct from 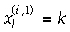,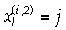. Collectively, the genotypes are represented by *X*.

Our method makes use of a computationally intensive approach, Markov chain Monte Carlo (MCMC), to perform inference. MCMC proceeds by starting from an initial arbitrary configuration of parameter values, and iteratively *updating* subsets of the parameters to new values, conditional on the data and the current values of the other parameters. In a single iteration of the algorithm, all of the parameters are updated once. For example, in the no-admixture model, structure starts with a random configuration of *Z*. In a single iteration, *P* is updated conditional on *X* and *Z,* and *Z* is updated conditional on *X* and *P*. The updates are constructed in such a way that after many iterations, the algorithm should converge to the posterior distribution of all of the parameters given the data. Practical issues related to convergence of the algorithm and interpretation of its results are discussed by Pritchard *et al*. (2000) and Falush *et al*. (2003b).

In order to extend structure to dominant markers, we now assume that there are observations X*, which provide partial information about the diploid genotypes *X* for the entire data set. We then introduce a new update step into the algorithm, which updates *X* based on the probability of all possible genotypes, conditional on X*, *P* and *Z.* All of the other updates can then proceed as usual, but conditional on the (current) imputed values of *X.* Specifically, we assume that at a subset of the loci, a single (known) allele *r* is recessive with respect to all of the others; this recessive allele will typically correspond to an allele copy that yields no PCR product. Any other alleles are assumed to be codominant with each other. So for example, a single AFLP observation consists of presence or absence of a band on a gel. In this case, absence of a band is the recessive state and the underlying genotype is ambiguous when a band is observed. For microsatellites, null alleles due to allele-specific PCR failure will be recessive to all other alleles at a locus.

In diploids, the genotype is ambiguous when a single allele *j,* different than the recessive allele *r,* is observed at a particular locus (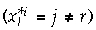). In this case, it is possible either that both allele copies at the locus are of type *j*, or that one of them is of type *r*. Using the standard structure assumption of conditional independence of the genotypes given *Z* (i.e. loosely speaking that there is Hardy–Weinberg equilibrium within populations) we have
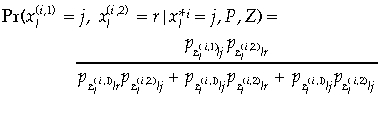

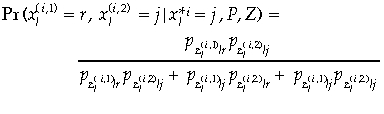
and
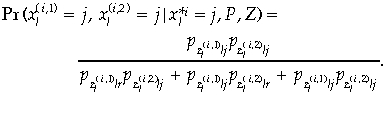


In each iteration, one of these genotypes is chosen at random according to its probability.

For organisms with ploidy greater than two, genotypic ambiguity may exist even if all alleles are codominant with each other. In triploids for example, if two alleles are observed at a locus, then either of them may be represented by two allele copies. We have implemented an algorithm that generates appropriate genotypes given both this ambiguity and (optionally) the ambiguity caused by recessive nulls, allowing dominant markers to be used. A trial genotype of individual *i* at locus *l* is generated by assigning each allele copy *a* independently, with probabilities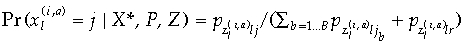, where *j*_1_…*j*_*B*_ are the *B* different codominant alleles observed at that locus and *r* is the recessive null allele (if applicable). Not all of the genotypes generated in this way will be consistent with the observed data; some will lack one or more of the observed alleles. We handle this difficulty by generating new trial genotypes until a consistent one is obtained; the resulting algorithm is inefficient computationally but is applicable for all ploidy levels.

Given an imputed value of *X*, all of the other updates can be performed as before. In the simplest case (i.e. ignoring updates of optional parameters such as the admixture proportions *Q*, described in Pritchard *et al*. 2000 and Falush *et al*. 2003b), the updates proceed as follows:

1. Update(*Z* | *P*, *X*)
2. Update(*X* | X*, *P*, *Z*)
3. Update(*P* | *X*, *Z*).

In order to initialize the algorithm in iteration 1, the Zs are first estimated randomly. For example, in the no-admixture model individuals are assigned randomly to each of the *K* populations with probability 1/*K*. The *Xs* are then initialized by ignoring any genotypic ambiguity, i.e. 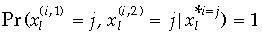. *P* can then be initialized using the normal update.

## Model validation

We validated the new model feature by using computer simulation to generate closely related populations. First, ancestral population frequencies were generated at 500 bi-allelic loci, with gene frequencies generated from a beta distribution (the bi-allelic version of a Dirichlet distribution) with parameter λ = 1 (see, e.g. Devlin & Roeder 1999; Falush *et al*. 2003b). Two populations were then generated with beta-distributed gene frequencies that were independently perturbed from the ancestral gene frequencies, assuming a value of genetic drift *F* = 0.1 for each one. Two hundred individuals were generated such that a known proportion *q*_*i*_ of individual *i*'s ancestry was from the first population and 1 − *q*_*i*_ from the second, where *i* = 1, …, 200. The *q*s were chosen according to a beta distribution with parameter α = 0.1. Thus, the data are generated according to a model that is very similar to the model assumed by structure to perform inference.

**Fig. 1:**
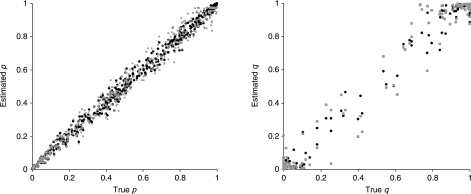
Comparison of estimated gene frequencies *p* and admixture proportions *q* with true values, for simulated data from two closely related populations. Black points show estimates for codominant markers. Grey points show estimates where allele 1 is dominant over allele 0. *p* represents the frequency of allele 1.

The data were handled in two different ways. In the first, the full genotypes were input into structure, so that the alleles were codominant. In the second, some information was removed from the data by making the allele designated 0 be recessive to allele 1; the diploid genotype 11 is then indistinguishable from 01 or 10; these three genotypes are all entered as 11. In both cases, we used structure assuming the correct model of dominance.

structure estimated both the ancestry gene frequencies *p* in the two populations and the ancestry proportion *q* of each individual (Fig. 1). As would be expected, the gene frequency estimates are more accurately estimated when the alleles are codominant, especially when the frequency of allele 1 is high. However, the difference is not all that large. Further, in this example, the mean squared error in estimating the ancestry proportions *q* is actually slightly higher when the alleles are codominant than when 0 is recessive, showing that the genotypic ambiguity caused by recessive alleles does not impose a substantial penalty on the quality of the inference, as long as it is handled correctly.

## Example

Normal and dwarf forms of the whitefish *Coregonus clupeaformis* coexist in several lakes in Canada. Campbell & Bernatchez (2004) typed 440 AFLP loci from 23 normal and 24 dwarf fish from Cliff Lake. We used structure to investigate possible gene flow between the two ecotypes.

**Fig. 2:**
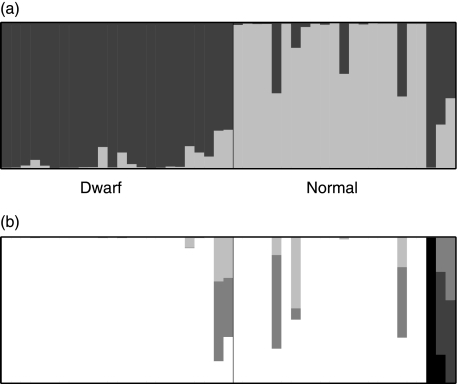
Ancestry of whitefish from Cliff Lake estimated using structure. (a) Ancestry estimates based on naïve clustering. Ancestry from the inferred dwarf and normal gene pools are shown in dark and light grey, respectively. (b) Likely ancestry of each individual, estimated using phenotypic information. White: full ancestry from observed ecotype. Light grey: one great grandparent with other ecotype. Grey: one grand parent with other ecotype. Dark grey: one parent with other ecotype. Black: both parents from other ecotype. See text for model details.

We first used the admixture model and performed clustering not using any population of origin information, under the *F* model, which assumes that the allele frequencies in the two populations are correlated. In view of the small number of individuals sampled, we fixed the number of populations to *K* = 2. We ran structure for 10 000 iterations after a burn-in of 1000 iterations. The divergence between populations estimated using the *F* model (analogous to *F*_ST_, Falush *et al*. 2003b), with a single value of *F* estimated was 0.25, implying that the two populations were quite strongly differentiated from each other before admixture occurred. The clustering correlated strongly with ecotype and most individuals were estimated to have almost all of their ancestry from a single population (Fig. 2a). However, there were several individuals who apparently had mixed ancestry, and one normal whitefish with a dwarf-like genotype.

In order to assess the statistical support for evidence of gene flow between the two groups, we used the USEPOPINFO option (Pritchard *et al*. 2000). This model assumes that most individuals classified as having a particular ecotype have pure ancestry from that type but that a small proportion of individuals may have a proportion of ancestry from the alternative ecotype. We set GENSBACK = 3, so that each individual could be of the alternative ecotype (i.e. they are misclassified) or have one parent, grandparent or great-grandparent with the alternative ecotype. In order to ensure that there is strong statistical support for any inference of mixed ancestry, we further set MIGRPRIOR = 0.001, implying that the prior probability that an individual has pure ancestry from its predefined ecotype is 0.999. Two dwarf and five normal whitefish showed strong evidence for mixed ancestry, with less than 50% posterior probability of having pure ancestry from the designated ecotype (Fig. 1b). These individuals are classified as hybrids with high probability, but there is some uncertainty remaining about how many generations ago the admixture occurred. One further whitefish shows a greater than 99% probability of having a dwarf genotype, despite being classified as normal. This individual presumably represents a classification error. The individuals who were classified as likely hybrids also showed the highest estimated admixture, implying that the two analyses are concordant (as were results for different runs with the same model, using different random starting points).

## Discussion

We have presented a simple approach that allows structure to handle dominant markers such as AFLPs and also genotype ambiguity for codominant markers in polyploids.

One common practical problem with otherwise codominant markers such as microsatellites is the presence of null alleles for which no PCR product is obtained. In principle, these can be handled by the model we introduce here, by treating such alleles as recessive. In practice, however, the model includes a number of assumptions that may not hold, including that the alleles that drop out do so consistently, e.g. due to mutations in the PCR primer binding site, rather than to variation in experimental conditions or low concentrations of DNA in a particular sample. In addition, the user must decide whether genotypes where no product is observed are homozygous for the null allele (in which case they should be recorded as such), or to other reasons such as low quality DNA (in which case they should be recorded as missing data; see Brookfield 1996 for relevant discussion). Finally, we note that inbreeding within populations may cause structure to infer a high proportion of null alleles, although it is unclear whether this might have a detrimental effect on inference for data sets on inbred populations, since structure currently assumes no inbreeding. In principle, individual-specific inbreeding coefficients could be estimated to allow for this. The approach taken here could also be extended to allow explicitly for genotyping errors, or allelic dropout due to low quality DNA. However, we have not attempted these extensions here.

The extensions described here will be particularly useful for researchers who would like to make inferences on patterns of gene flow in natural populations, but have only relatively limited resources for genotyping. We have shown that by using 440 AFLP markers, it is possible detect whitefish with mixed ancestry from two different ecotypes, even when the admixture occurred two or three generations ago, so that three-quarters or more of the ancestry of the fish in question comes from a single population. Larger numbers of markers, in combination with linkage information, would allow yet more detailed inferences.
